# Honey bee genetic resistance outperforms a cold-storage induced halt in brood production to control mites and viruses

**DOI:** 10.1038/s41598-026-44701-3

**Published:** 2026-03-21

**Authors:** William G. Meikle, Milagra Weiss, Daniela Adjaye, Vincent A. Ricigliano

**Affiliations:** 1 Carl Hayden Bee Research Center, USDA-ARS, Tucson, AZ 85719 USA; 2 Honey Bee Breeding, Genetics, and Physiology Research, USDA-ARS, Baton Rouge, LA 70820 USA; 3 Invasive Species and Pollinator Health Research Unit, USDA-ARS, Davis, CA 95616 USA

**Keywords:** Colony health, Bee stock, Pol-line, Varroa, Deformed Wing Virus, Vitellogenin, Thermoregulation, Hive CO_2_ concentration, Ecology, Ecology, Zoology

## Abstract

*Varroa destructor* mites seriously threaten honey bees by spreading viruses like Deformed Wing Virus (DWV) and contributing to global colony losses. Growing resistance to widely-applied miticides highlights the urgent need for sustainable mite control methods. This study evaluated the impact of a cold storage strategy to decrease bee brood production, and increase mite treatment efficacy, in a commercial Italian bee stock and mite-resistant Russian and Pol-line bee stocks, from prior to cold storage in August until the start of the commercial pollination season the following February. For each year of two years, thirty new bee colonies (10 colonies per stock) were either placed in cold storage (5 °C, darkness, 18 days) starting mid August or left outdoors, and all hives subsequently treated with a thymol-based varroacide. Colony brood area, adult bee mass, hive weight, internal temperature and CO₂ levels were monitored during the experiments using periodic hive assessments as well as sensors. Honey bee workers were sampled at different points and evaluated for bee health biomarker gene expression (*vitellogenin*) as well as virus levels of *DWV-A* and *DWV-B*. Cold storage effectively halted brood production but differences in brood levels between groups disappeared within two months, with no long-term impact on population size, mite levels, virus loads, or daily hive weight change. Bee stock was the dominant factor influencing outcomes: mite susceptible Italian colonies had higher mite densities, higher DWV loads, lower *vitellogenin* expression and higher rates of hive weight loss than Russian or Pol-line colonies. In this study mite-resistant honey bee stocks offered more effective control, reducing mite loads by over 65% compared to the susceptible stock, across both years and both treatment groups of the study, and they have the potential to support honey bee health by reducing reliance on chemical treatments in beehives.

**Supplementary Information:**

The online version contains supplementary material available at 10.1038/s41598-026-44701-3.

## Introduction

Effective management of Varroa mite (*Varroa destructor* Anderson and Trueman) infestations in honey bee (*Apis mellifera* L.) colonies represents a significant operational challenge for commercial beekeepers. Varroa mites are costly to manage and, if uncontrolled, weaken colonies and spread viruses such as Deformed Wing Virus^1–3^. Resistance of Varroa mites to common miticides, such as fluvalinate, coumaphos and amitraz, has been reported^4^. Beyond resistance concerns, reduced reliance on chemical treatments is desirable since miticides are often found in high concentrations in colony matrices such as pollen and wax^5^, raising additional concerns about bee and human health. Mite control is particularly challenging in warm climates, with a long active season that encourages honey bee colonies to produce brood much of the year^6^. Varroa mites reproduce in bee brood, so a longer period of brood production results in a longer period of Varroa population growth. In addition, Varroa mites living within sealed brood cells are largely protected from miticides, so the more brood present in an infested colony during miticide application, the greater the proportion of the Varroa population protected from miticides^7^.

One approach to reducing the number of Varroa mites that are protected by the cap seal of mature brood is to prevent the queen from laying eggs, either by caging or removing her^7^, or by inducing her to stop oviposition by changing environmental conditions to simulate winter, such as cold and darkness^8^. Cold storage lasting 2–4 months is often used in the northern latitudes of the US as a way of protecting colonies against weather events from late fall until late January^9^. Cold storage was used here simply to halt oviposition by the queen and allow for emergence of remaining brood, a process which needs at most 3 weeks^10^. Once the existing brood has emerged, the protected space for Varroa will be reduced or eliminated and all remaining mites will belong to one of two sub-populations: (1) attached to adult worker bees (the “dispersal phase”); or (2) crawling on the surface of the wax comb^11^. These sub-populations are typically more vulnerable to miticide treatment^12,13^.

Miticides are not the only way to combat mites; honey bees can be selected for behaviors and genetics that maintain Varroa mite populations at lower levels^14,15^. Russian and Pol-line honey bee stocks, developed as part of USDA breeding programs, have shown resistance to Varroa mites^16,17^. In cage studies, Russian, Pol-line and Italian bee stocks responded differentially to viral infection and food preferences^18,19^, and Pol-line and Russian bees were shown to have longer adult lifespans, lower food consumption rates, and increased *vitellogenin* expression relative to Italian bees^20^. Field studies have also shown differences among these bee stocks. Pol-line and Russian colonies gained weight faster during the primary nectar and pollen flow season (June-September) and slower weight loss during the dearth season (October - February), as well as lower Varroa mite densities, compared to Italian colonies^21^.

The objective of this study was to evaluate a two-pronged strategy comprising a halt in brood production, or “brood break”, plus a miticide application, across the Pol-line, Russian and Italian honey bee stocks. The basic experimental design was to divide bee colonies into two groups, one group subjected to a cold storage treatment to induce a break in brood production, and the other group as a control. Both groups would be treated with a thymol-based miticide after the cold storage period and monitored prior to cold storage in August through winter until February, to observe short and longer-term effects. Bee colonies would be managed as they would be in a professional apiary with respect to inspections, feeding and other interventions. Thymol-based miticide was chosen because it is commonly used in the beekeeping industry^22^ and resistance has not been reported. This work adds to our previous research^10^ by exploring the effects of starting cold storage in August compared to early October, and by incorporating different bee stocks as a grouping factor, thus including a genetic component. Response variables include discrete data, such as colony population size, mite levels, and the expression of *vitellogenin* (Vg) and Deformed Wing Virus (hereafter “DWV”) –A and DWV-B, as well as continuous data, such as hive weight, temperature and internal CO_2_ concentration.

## Results

**Fig. 1:**
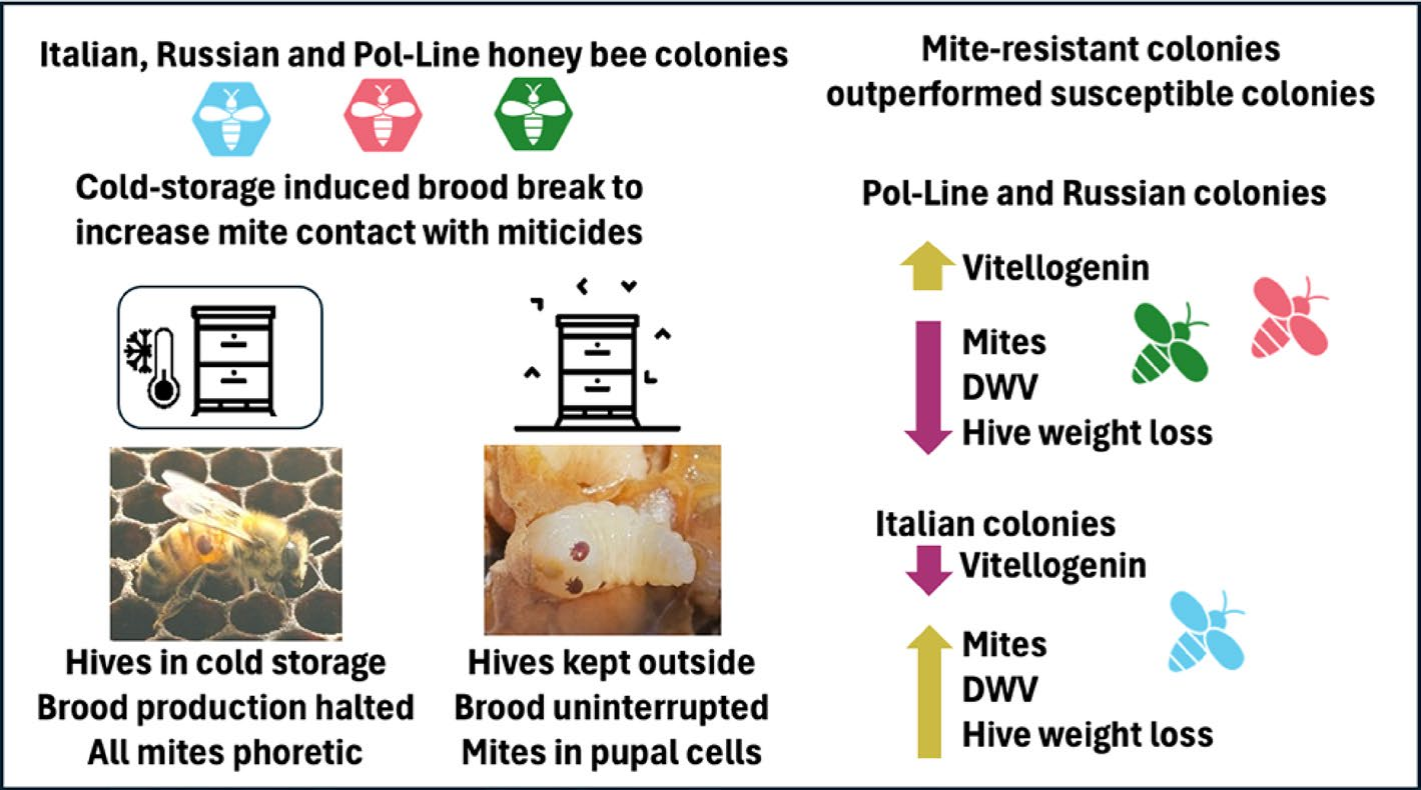
Graphical representation of experimental design and main results.

A brief summary of the experimental design and main results are shown (Fig. 1).

Experimental activities were conducted on similar timelines across the two experiments (see Table 1).

**Table 1:** Timeline of activities for the two experiments. Ranges of dates are shown because activities were often not conducted on exactly the same date.

Action	Dates
Install hive scales, temperature and CO_2_ sensors	1 Aug
Hive evaluations, Bee samples for gene expression, Bee samples for mite loads, Mite fall measurement	12–17 Aug
Cold storage	19–21 Aug to 6–8 Sep
Miticide treatments (weekly for 3 wks)	9–12 Sep to 24–28 Sep
Hive evaluations	27 Sep to 2 Oct
Mite fall measurement	24–27 Oct
Hive evaluations	12–14 Nov
Bee samples for gene expression	21–28 Nov
Bee samples for mite loads	30 Jan
Bee samples for gene expression	15–18 Feb
Hive evaluations	18–22 Feb
Remove hive scales and sensors	22 Feb

### Discrete colony measures

A summary of discrete colony measures is provided (Supplementary Table S1).

#### Colony population measures

**Fig. 2:**
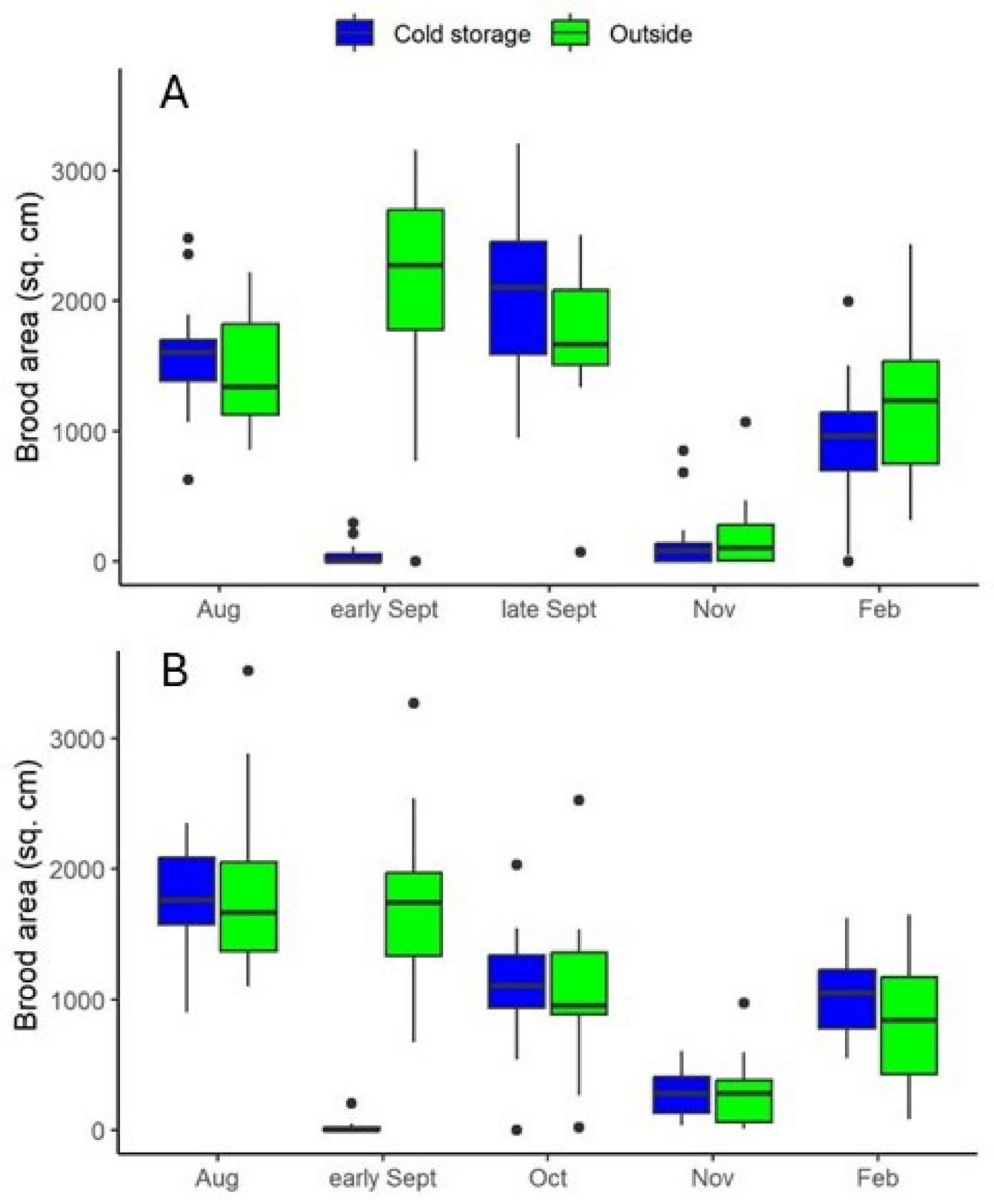
Surface area of total capped brood per colony across two groups: bee colonies put in cold storage (“Cold storage”) and bee colonies kept in an outdoors apiary (“Outside”). (**A**) 2023-24; (**B**) 2024-25. Hives were removed from cold storage just prior to the early September assessment. Colonies placed in cold storage had significantly less brood than hives kept outside at the early September assessment; no other comparisons were significant.

In a repeated measures analysis, adult bee masses were not different either between treatment groups or among bee stocks (Supplementary Table S2). Neither brood nor log transformed brood was normally distributed, so nonparametric statistics were conducted. Italian colonies had significantly more capped brood than either Pol-line or Russian colonies prior to treatment but no differences in brood level were observed among bee stocks within cold storage treatment group after treatment (Supplementary Table S3). Post cold storage, colonies had negligible amounts of worker brood and no drone brood. Post miticide treatment, colonies put into cold storage had significantly less brood in the 1 st sampling occasion than colonies kept outside, but from the 2nd sampling occasion onward no differences were observed (Fig. 2). Across the 60 colonies of the study, 13 colonies either superseded or lost their queens at some point and were either requeened or removed from the study. Six of those colonies (three Russian and three Italian) were in the cold storage treatment compared to 7 control colonies (four Russian, two Italian and a Pol-line) indicating no negative effect of cold storage treatment on queen health. Thus cold storage treatment itself had no observed long-term effect on brood production or queen health.

#### Gene expression

**Fig. 3:**
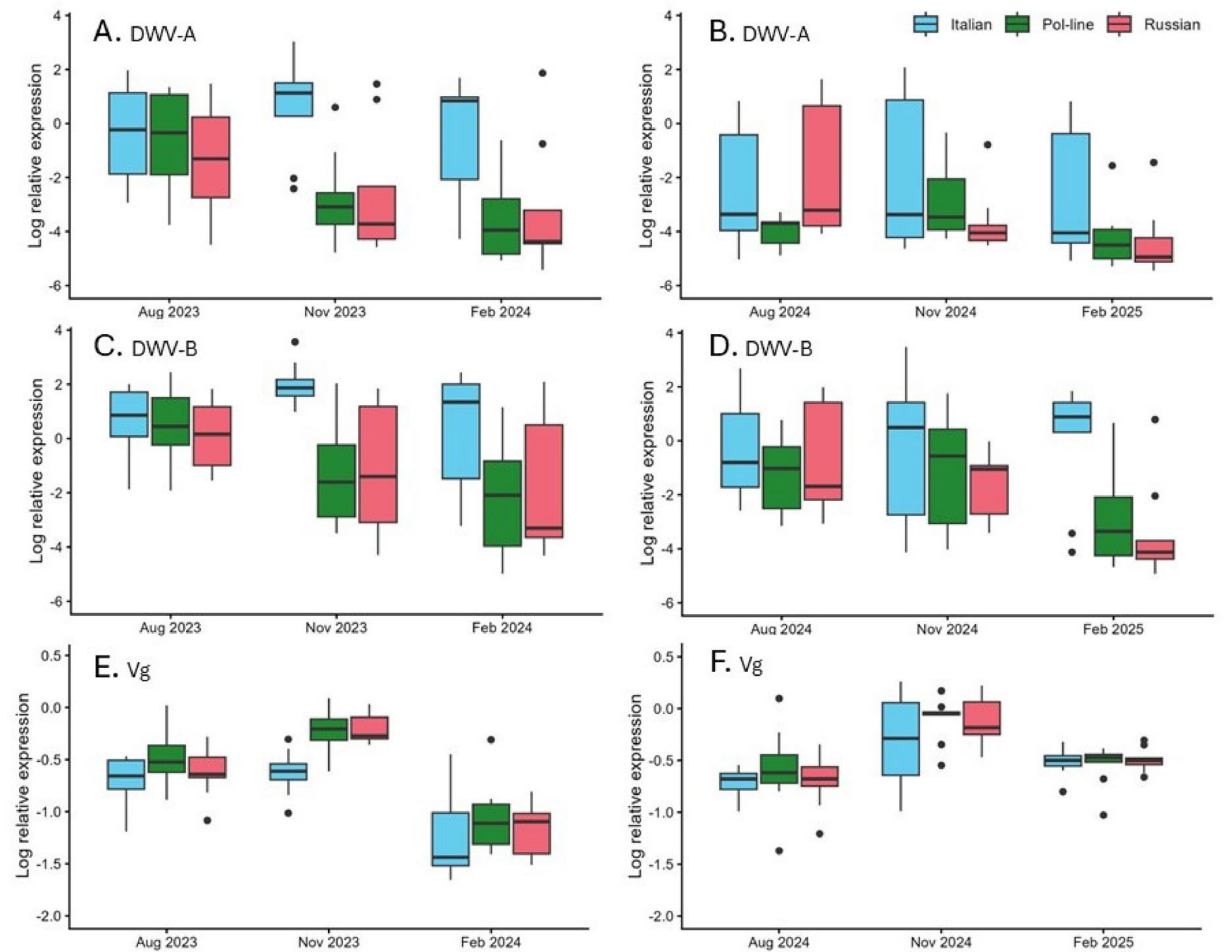
Gene expression among adult worker bees during the two years of the study. (**A**, **C**, **E**) on the left: results from 2023-24; (**B**, **D**, **F**) on the right: results from 2024-25. Italian stock colonies had significantly higher DWV-A and DWV-B titers, and significantly lower vitellogenin titers, than Pol-line or Russian colonies for both the November and February samples, and Pol-line and Russian colonies were not different between themselves. No comparisons pre-treatment in August were significant.

Neither raw or transformed data for DWV-A or DWV-B gene expression were normally distributed and so were analyzed using nonparametric tests. Comparisons of DWV-A and DWV-B gene expression were conducted with treatment and bee stock as main effects, and as bee stock within treatment group, for each of the three sampling occasions: August (pre-treatment); November and February. Bee stock after treatment was significant, with Italian colonies having significantly higher viral expression post treatment and post winter than Pol-line (*P* = 0.039 for both samples) or Russian colonies (*P* = 0.031 and 0.023, respectively) for DWV-A (Fig. 3; Supplementary Table S4). Similar results were observed for DWV-B, with Italian colonies having higher viral expression post treatment and post winter than Pol-line (*P* = 0.039 and 0.004, respectively) or Russian colonies (*P* = 0.020 and 0.007, respectively). Pol-line and Russian colonies were not significantly different at any point. The years of the experiment, 2023 and 2024 were significantly different with respect to the pre-treatment sample but not in later samples (Supplementary Table S5). Cold storage treatment groups did not show significant differences. When bee stock was nested within treatment group, to investigate whether treatment changed the relationship among the different bee stocks, few significant differences were observed, due at least partly to the low number of observations per sub-group (Supplementary Table S6).

Transformed (arcsine of the square root) gene expression data for Vg were sufficiently normally distributed for the application of repeated-measures MANOVA (Supplementary Table S7). Vg expression was significantly affected by bee stock and year of study as main factors, and post hoc contrasts showed that Italian bees had significantly less Vg expression than either Pol-line (*P* = 0.003) or Russian colonies (*P* = 0.023). In this study, bees in the 2023-24 experiment had lower Vg expression than did bees in the 2024-25 experiment.

#### Varroa mite levels

**Fig. 4:**
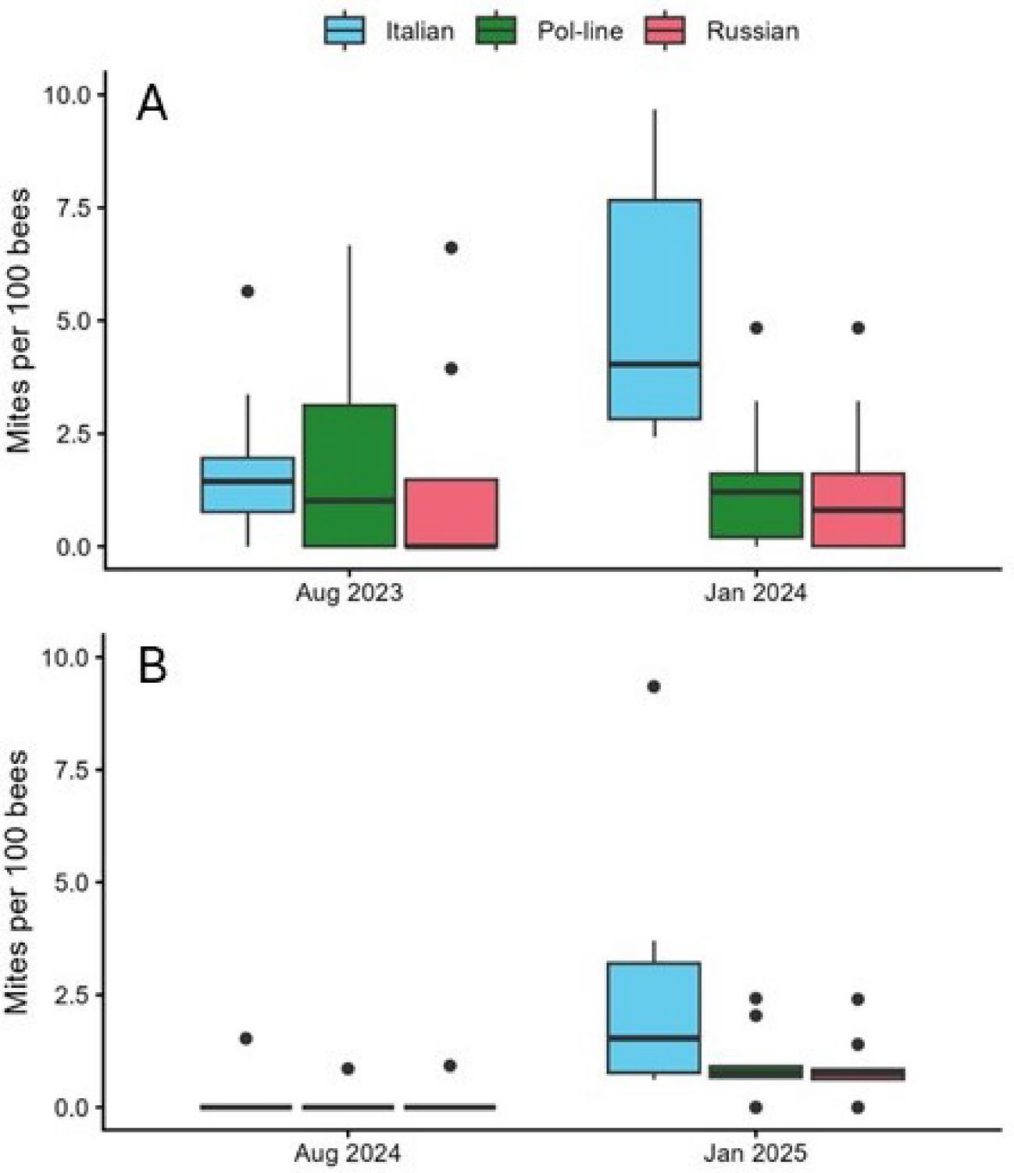
Mites per 100 bees resulting from alcohol washes of samples of adult worker bees. (**A**) 2023-24; (**B**) 2024-25. Overall, Italian colonies had significantly higher mite loads than Pol-line or Russian colonies, which were not different between themselves.

Mite alcohol wash data either raw or transformed, were not normally distributed so analyses were restricted to nonparameteric tests (Supplementary Information Table S8). As with the gene expression data, only bee stock was a significant factor in those analyses, both for post-treatment (January) mite levels, and the difference between the pre-treatment and post winter samples (Fig. 4). Italian colonies had the highest mite levels overall (including hives that went into cold storage and hives that did not) with an average ± s.e. of 3.7 ± 0.7 mites per 100 bees, compared to Pol-line (1.2 ± 0.3 mites per 100 bees) and Russian colonies (1.1 ± 0.3 mites per 100 bees). Italian colonies also had the largest increase in mite densities (2.9 ± 0.8 mites per 100 bees) between pre-treatment and post-winter compared to Pol-line and Russian colonies (0.1 ± 0.5 and 0.3 ± 0.4 mites per 100 bees, respectively). DWV-A and DWV-B levels were strongly correlated with mite loads in August (*r* = 0.49 and 0.50, respectively, with both *P* = 0.0001) but the relationship was weaker in February for DWV-A (*r* = 0.31, *P* = 0.029) and not significant for DWV-B (*r* = 0.26, *P* = 0.06).

No differences were observed with respect to mite fall on sticky boards were observed either between treatment groups, among bee stocks, or among treatment group x bee stock subgroups (Supplementary Information Table S9).

### Continuous hive measures

#### Daily hive weight change

**Fig. 5:**
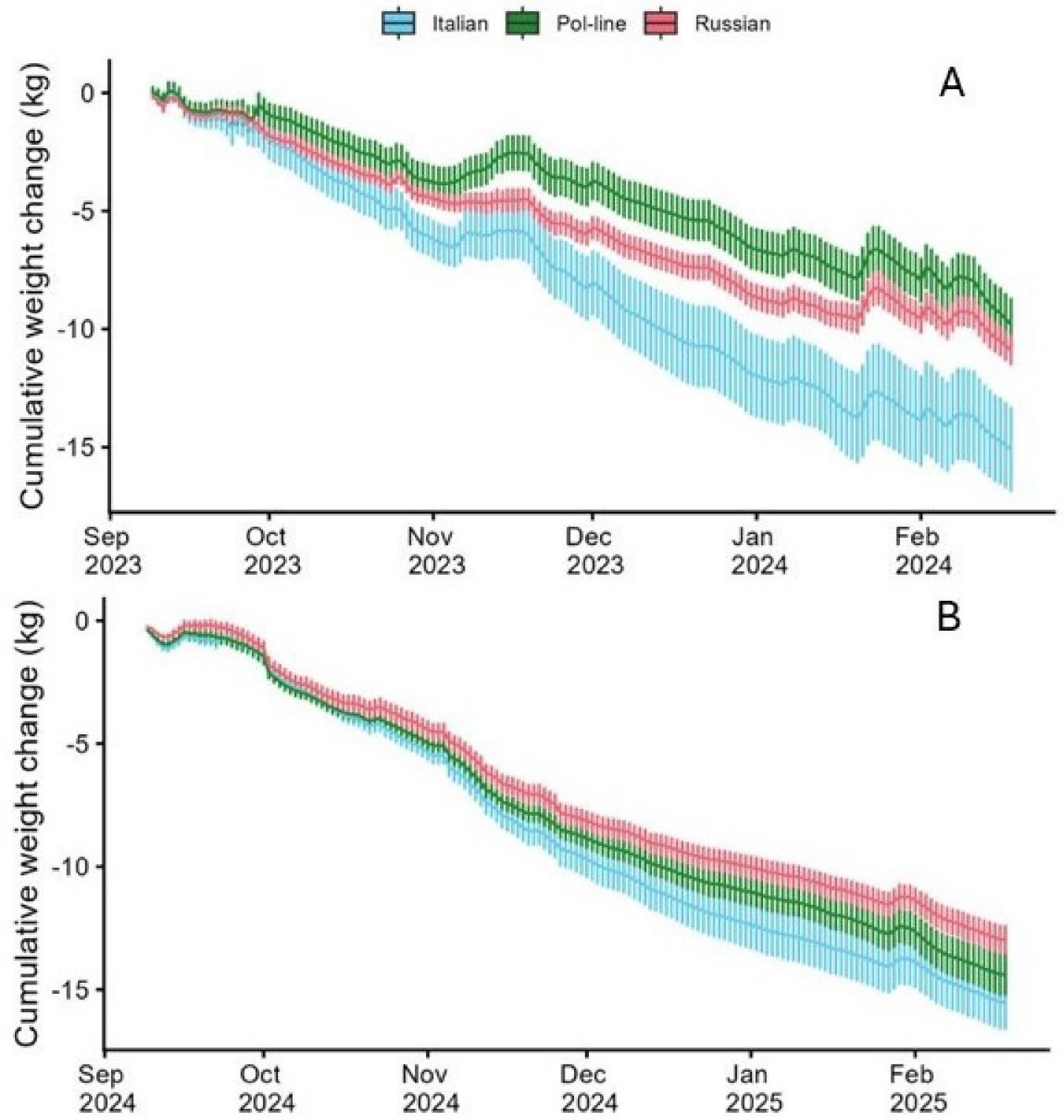
Average cumulative weight change (± s.e.) from September (immediately post cold storage treatment) to mid February. (**A**) 2023-24; (**B**) 2024-25. Overall, Italian colonies had significantly higher weight loss per day than Pol-line or Russian colonies, which were not different between themselves.

Daily hive weight change was significantly affected by both bee stock and the year of the study, but not by treatment (Fig. 5; Supplementary Information Table S10). Hives lost a cumulative average of 12.36 ± 0.78 kg during 2023-24 experiment, and 14.33 ± 0.54 kg during the 2024-25 experiment. On a daily basis, hives in 2024 lost about 13 g more per day on average than hives in 2023. Across both experiments, Italian hives lost on average of 15.30 ± 0.21 kg, Pol-line hives lost 12.09 ± 2.29 kg and Russian hives lost 11.93 ± 1.04 kg. On a daily basis, hives with Italian colonies lost an average of about 20 g more per day than Pol-line hives (*P* = 0.001) and about 23 g more per day than Russian bees (*P* < 0.001), while Pol-line and Russian hives were not significantly different between themselves.

#### Daily hive temperature

**Fig. 6:**
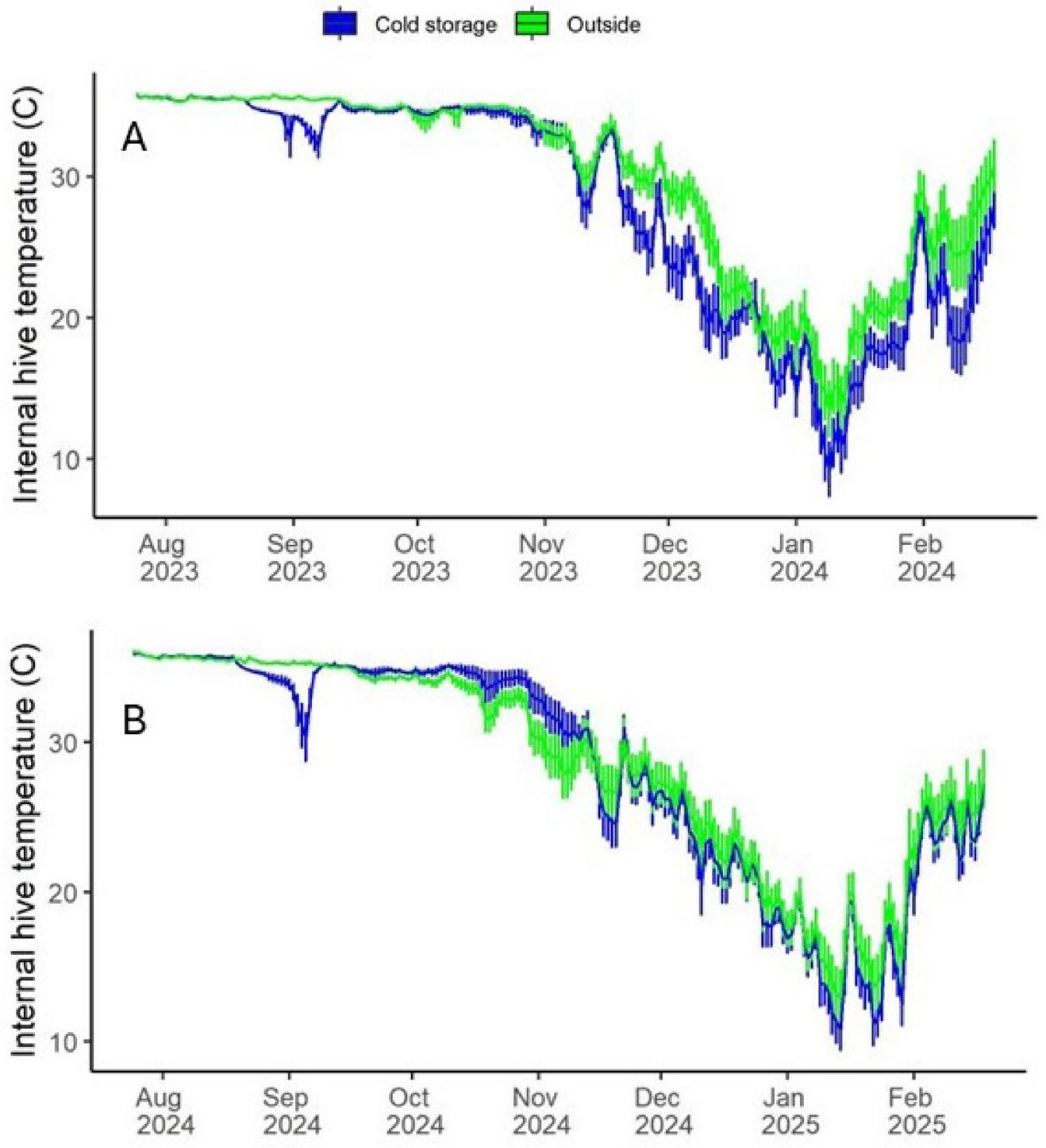
Average hive temperature from mid August (pre cold storage treatment) until mid February. (**A**) 2023-24; (**B**) 2024-25.

Log daily average temperatures differed between the two treatment groups, with hives subjected to cold storage treatment showing significantly lower average temperatures overall (Fig. 6; Supplemental Information Table S11). Average temperatures for hives put in cold storage across both years was about 26.3 ± 0.1 °C (this includes winter, when little brood was present) while hives kept outside averaged 27.6 ± 0.1 °C. Average temperature amplitudes were significantly affected by both treatment and the year of study. Hives subjected to the cold storage treatment had more variable temperature, with daily amplitudes averaging 2.7 ± 0.1 °C on average compared to hives kept outside, which averaged 1.9 ± 0.1 °C. Temperature amplitudes of hives monitored in the 2023-24 experiment averaged 1.9 ± 0.1 °C while those in 2024-25 averaged 2.7 ± 0.1 °C, again possibly due simply to different amounts of brood between the two years: post treatment, average amount of brood across both treatments and all bee stocks in 2023-24 was 1047 ± 341 cm^2^ while in 2024-25 the average was 757 ± 172 cm^2^.

#### Daily hive CO_2_ concentration

**Fig. 7:**
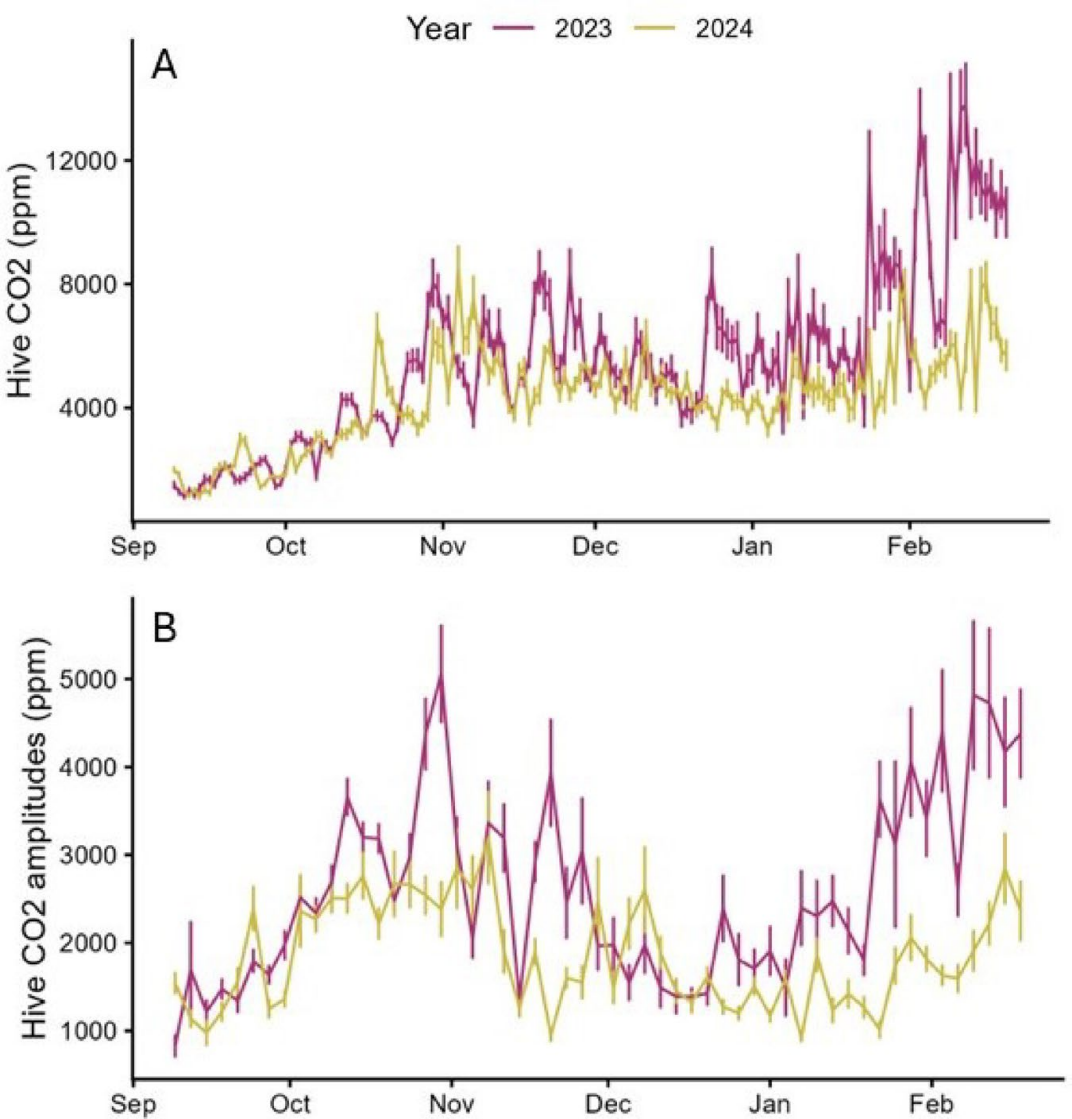
Average hive CO_2_ concentration, across bee stock and cold storage treatment groups, from 9 September (post cold storage treatment) until mid February. (**A**) Average values ± s.e.; (**B**) Amplitude values ± s.e.

Hive CO_2_ concentrations were not different between treatment groups or among bee stocks but did differ between the two years of the study (Fig. 7; Supplemental Information Table S12). Average CO_2_ concentrations were 5502 ± 60 ppm in 2023-24 and 4247 ± 34 ppm in 2024-25, and average daily amplitudes were 2596 ± 58 ppm in 2023-24 and 1827 ± 36 in 2024-25.

## Discussion

The main objective of this research was to evaluate the efficacy of a late summer, 3-week long cold storage treatment as part of a Varroa mite control strategy and to compare the effects of that treatment across different bee stocks. The purpose of cold storage treatment in this study was only to induce a cessation in brood production and thus restrict the mite population to either the exposed surface of wax comb or on adult bees^11^ where they increased exposure to miticides. Such halts in brood production can also be induced by caging or removing the queen, but these methods are riskier for the queen and require more work per hive than stopping oviposition through environmental factors like cold and darkness. The timing, duration and purpose of the cold storage period in this study differ from those of overwintering cold storage of bee colonies commonly practiced in the US, which typically start in late fall, last at least 2 months and are intended to protect colonies from outdoor weather threats.

Response variables in this study consisted of colony-level estimates of bee and mite population sizes, and DWV and *vitellogenin* gene expression as well as continuous data on hive weight, temperature and internal CO_2_ concentration. Cold storage itself had no measurable effect on adult bee population sizes, daily hive weight change, within-hive CO_2_ concentration or on the expression of DWV or *vitellogenin*. Cold storage halted brood production, as was expected, but the effect was short-lived. By mid November, there were no significant differences in terms of brood between colonies that had been subjected to cold storage and those that had not.

These experiments build on the results of a previous study that used the same bee stock throughout but included the presence and absence of the post-storage mite treatment^10^. In that previous work, cold storage treatment did not have a significant effect on mite levels and several hypotheses were proposed to explain that lack of success: (1) local environmental conditions after cold storage, i.e. little or no forage, were poor for colony recovery (so colony health of both treated and untreated colonies was comparatively low but for different reasons); (2) some parameters, such as the timing (in this case starting early October) and duration (20–22 days) needed to be changed to improve efficacy; and (3) Varroa levels prior to storage were simply too high for successful treatment in this manner (28% of the colonies in that study had Varroa infestations > treatment threshold of 3 mites/100 bees^12^. Changing the time of year of the storage treatment, as done here, would address parts of both 1) and 2). If colonies were placed in cold storage earlier, they would exit cold storage earlier when some forage would likely still be available, and they would also have more time to recover before the fall and winter forage dearth. While colonies in the previous study had sufficient honey reserves for the duration of the experiments, and were fed protein patty immediately after cold storage, the availability of some forage in the environment might induce colonies to increase brood production and thus enter the winter with larger bee populations. Regarding hypothesis 3), that Varroa levels were simply too high for control in the earlier study would be less likely in these experiments – only 13% of the colonies in in this study had mite levels exceeding the threshold for late summer/early fall application of 3 mites/100 bees.

This study concerned the exposure, or not, to 18 d cold storage starting in August, across three different bee stocks, followed by a September mite treatment and colony monitoring until the start of the commercial pollination season the following February^23^, about 180 d later. This was a logical period to evaluate treatment from a commercial beekeeping perspective in the western U.S.A. As noted above, the cold storage strategy did not have a significant effect on either mite drop onto sticky boards or dispersal phase mite densities by the end of the study. Mite loads (mites/100 bees) at the end of the study, across all bee stocks and both years, were 1.73 ± 0.36 for colonies placed in cold storage and 2.14 ± 0.52 for control colonies. In contrast, several previous studies have reported success in lowering Varroa mite levels in bee colonies by combining an artificially-induced brood break, by either cold storage or caging the queen, followed by a miticide treatment^22,24–26^. Successful outcomes were achieved with thymol^22^, potassium salt of hop beta acids^26^, amitraz^26^, and oxalic acid^24,25^, although in a comparison of miticides oxalic acid was found ineffective^26^. Those studies were conducted for shorter periods (31–144 d) and at a different time of year (between Mar. and July) than this study, which may at least partly explain the different results. Another study evaluated caging queens followed by an oxalic acid treatment, over 66 d starting in Sept. but did not report success in reducing mite loads^27^.

Most colonies in this study were of mite resistant bee stocks resulting in low mite densities overall, with no colonies exceeding 10 mites/100 bees. This is different from the previous study, in which only a nonresistant bee stock was used and several alcohol wash samples exceeded 15 mites/100 bees^10^. At the end of this study, mite loads among bee stocks were significantly different- across both treatment groups and both years, Italian colonies had, on average, 3.65 ± 0.74 mites/100 bees, compared to 1.20 ± 0.29 for Pol-line colonies and 1.09 ± 0.31 for Russian colonies. Thus, resistant bee stock reduced mite loads over 6 months on average by over 65% compared to non-resistant stock. The analyses of long-term treatment effects on bee colonies (using continuous data on hive weight and internal conditions), and indirect effects of Varroa infestation (i.e. virus and vitellogenin levels) as well as data on Varroa levels and colony size, supported the positive effect of bee stock and the lack of cold storage strategy effect.

As has been observed previously using the same bee stocks^21^, Italian colonies produced more brood, had higher levels of mites, and lost weight faster than Pol-line and Russian colonies. Pol-line and Russian colonies in this study also had significantly lower levels of DWV-A and DWV-B. DWV-A levels were correlated with mite levels for both the August and January samples, but while DWV-B levels were correlated with mite levels in August, those in February were not, suggesting a more complex relationship among bee stock, Varroa mites and DWV in general. The expression of *vitellogenin*, an important nutritional storage protein associated with longevity^28^, was significantly lower among Italian colonies, which has been observed previously in cage studies^20^. However, *vitellogenin* expression is also affected by forage conditions^28,29^: for example, in the previous study *vitellogenin* expression among bees was much higher in a year of high rainfall, and thus a remarkably high availability of forage, than in a year of low rainfall^10^. Rainfall in this study was not as different between the years of this study, with about 260 mm falling in 2023 and 293 mm in 2024, but the distribution was different between years, with about 76 mm falling in the first four months of 2023 and 134 mm falling during that same period in 2024^30^. The heavier rainfall in 2024 stimulated local plant blooms in spring and early summer. Colonies in 2024 therefore had better forage opportunities, supporting the link with *vitellogenin* expression. Queen loss or supersedure after early September (before that problematic colonies were simply removed from the experiments) was higher among Russian colonies (7 out of 20 colonies) than among Italian (5) or Pol-line bees (1).

Three weeks in cold storage treatment was effective at inducing a brood break, as previous work had shown most or all brood had emerged after about two weeks in cold storage^10^, with no consistent adverse effects. The only long-term effect of cold storage was a somewhat lower hive internal temperature (please see below). This is remarkable considering bee colonies were moved from ambient conditions, i.e. average daily temperatures of about 32°C^30^ and daylengths exceeding 12 h, into the cold storage unit for three weeks with a temperature of 5 °C and total darkness, and then moved back into warm, sunny ambient conditions. Brood breaks are often induced by caging the queen^22,24,25,27^, although problems with colony mortality have been reported with that method^27^. Results reported here indicate that while the cold storage strategy as applied here did not affect Varroa populations, cold storage even in summer can be an effective method to induce a brood break.

Hive temperature and CO_2_ concentration are a function of colony-level behavior^31^. In this study continuous temperature and CO_2_ monitoring revealed group differences not detected with other colony measures. Internal hive temperature averages and amplitudes have been correlated with the presence of brood^32^, and daily hive temperature patterns have been found sensitive to factors such as sublethal pesticide exposure when other parameters, such as colony size, were not^33^. The lower averages and higher amplitudes in hives subjected to cold storage may have partly resulted from low brood levels immediately after cold storage, but average temperatures were consistently lower in the cold storage treatment group for several months. It may be that cold storage itself affected colony thermoregulation, but a simpler explanation may be that some colonies in the cold storage treatment moved the brood nest, where temperatures are carefully maintained at 34–35°C^34^ to a location in the hive further from the sensor (sensors were placed in the same location in each hive). Hive temperatures further from the brood cluster are lower and more variable in the fall and winter, reflecting a greater influence of outside conditions on the sensor^32^.

In-hive CO_2_ concentration was not affected by treatment or bee stock in this study but was higher and more variable in 2023 compared to 2024. Like hive temperature, hive CO_2_ concentration has been found to exhibit strong daily cycles driven by light^31^ but the 18 d of constant darkness of cold storage had no measurable effect on CO_2_ average values or daily amplitudes. Since adult bees both produce CO_2_ and control hive ventilation, adult bee population size was expected to be related to within-hive CO_2_ concentration, but there were no significant differences in adult bee mass between years or treatment groups or among bee stocks. The factors driving CO_2_ concentration in bee hives are not well understood; for example, average CO_2_ concentrations have been found significantly higher in smaller colonies than larger ones^33,35^, in better ventilated hives than less ventilated hives^36^, in colonies fed syrup with 5 parts per billion imidacloprid than in control colonies^33^, and in Pol-line colonies than in Italian or Russian colonies^21^. The interaction of cold storage treatment and bee stock was a significant factor in CO_2_ concentration.

This work represents the culmination of a four-year, two-part project on the impact and efficacy of a cold-storage induced brood break, followed by a thymol-based miticide, as a Varroa mite control strategy. That cold storage strategy was not effective, whether hives were placed in cold storage in August or October. However, cold storage in August did not have any lasting effects on the bee colonies themselves. While the colonies in cold storage ceased producing brood, within two months there was no detectable difference between those colonies and the ones that remained in field conditions. The second major observation was the efficacy of bee stock for reducing mite and virus levels. Bee stock proved much more effective than the cold storage strategy tested here, as shown by the lower mite populations and DWV titers in mite resistant stock. As such, genetic resistance is likely to become a crucial management component as evidence mounts that mites are evolving resistance to commonly used miticides^4,37^. Combining genetic resistance against *Varroa* with emerging technologies such as RNAi targeting mites and viruses could form the basis for integrated pest management strategies that are no longer reliant on chemical inputs into beehives and the environment^38,39^. Commercial beekeeping operations may find this work useful when balancing the costs of mite resistant queens against the costs of mite treatment.

## Materials and methods

Two experiments were conducted, the first from August 2023 – February 2024, and the second a year later with new colonies. In August of each year 30 honey bee colonies, comprising ten colonies of each of three bee stocks: Pol-line, Russian and Italian, were identified. The colonies had been established from single box colonies received in mid April. Bee colonies were housed in painted, two-box, 10-frame, wooden Langstroth hives (43.7 L capacity per box). The hives were located at the University of Arizona Red Rock Agricultural Station (GPS coordinates: 32.55, −111.35) where they were placed on stainless steel electronic scales (Tekfa model B-2418 and Avery Weigh-Tronix model BSAO1824-200, max. capacity: 100 kg, precision: ±20 g; operating temperature: −30 °C to 70 °C) and linked to dataloggers (Hobo UX120-006 M External Channel datalogger, Onset Computer Corporation, Bourne, MA) with weight recorded every 5 min. Temperature sensors (HOBO MX2201, resolution ± 0.04 °C, accuracy ± 0.5 °C) were attached to the center of the top bar on the middle frame in the bottom box and set to record every 5 min. Carbon dioxide probes (model GMP251, Vaisala Inc., Helsinki, Finland), calibrated for 0–20% concentrations, were placed on top of the center frames in the top box of each hive and linked to dataloggers (HOBO UX120-006 M) set to record every 5 min.

Hives were periodically assessed using a published protocol^40^ to determine adult bee mass and total capped brood area. Briefly, each hive was opened and each frame was lifted out sequentially, gently shaken to dislodge adult bees, photographed using a 16.3 megapixel digital camera (Canon Rebel SL1, Canon USA, Inc., Melville, NY), weighed on a portable scale (model EC15, OHaus Corp., Parsippany, NJ), and replaced in the hive. During the first assessment all hive components (i.e. lid, inner cover, box, bottom board, frames, entrance reducer) were also shaken free of bees and weighed to yield an initial mass of all hive components. The total weight of all hive components and frames was subtracted from the total hive weight recorded during the night pre-assessment, to calculate the adult bee mass. The area of sealed brood per frame was measured from photographs using ImageJ version 1.47 software (W. Rasband, National Institutes of Health, USA) or CombCount^41^, and frame values were summed to estimate colony values. Assessments took place in mid-August (pre-cold storage treatment period), late September (after the cold storage and mite treatments), mid-November (pre-winter period), and a final assessment in mid-February (post-winter period). Bee colonies were managed as they would be in a professional apiary, with care taken to perform the same procedures, e.g. feeding, on all hives and avoid bias. Queen presence was monitored at each assessment; all queens were marked with paint on the thorax, and any queens lost during the experiments were replaced by queens of the same bee stock. Colonies needing more than one re-queening were removed from the study. In November and at the end of January all colonies were fed 200 g pollen patty, made at a ratio of 1:1:1 corbicular pollen (Great Lakes Bee Co.): granulated sugar: drivert sugar (Domino Foods, Yonkers, NY).

After the first assessment, the 10 colonies of each bee stock were ranked with respect to adult bee mass and then assigned to a treatment group, ensuring that average bee mass per colony was approximately equal between the two groups. Fifteen hives (3 bee stocks x 5 hives) were then moved into a cold storage unit (CSU) (30 m^3^ internal volume, with CO_2_ and temperature monitors, PolarKing, Fort Wayne, IN) at the USDA-ARS Carl Hayden Bee Research Center in Tucson AZ (GPS coordinates: 32.28, −110.94) in late-August for 18 d to induce a break in brood production. The CSU was set to 5 °C with a dehumidifier and a roof-mounted exhaust fan operating 5 min per hour for ventilation. Immediately after the 18 d hives were moved out of the CSU back to the Red Rock apiary, all colonies were treated with thymol miticide (Apiguard, Vita Bee Health, Basingstoke, UK) at 25 g/week for 3 weeks, as per manufacturer instructions for application in warm temperatures^42^. Three weeks was considered sufficient, as worker and drone brood are capped for about 14 d, so any cells would be exposed to miticide for at least part of the treatment period^43^. Thymol-based miticide was used to avoid potential issues with amitraz resistance^4^. Thereafter colonies were managed in an identical manner throughout the fall and winter, concluding the following February.

Samples of 100 adult bees were collected from the brood nest area of the hive in August (pre-treatment), in November and in late January for gene expression analysis, and an additional approximately 150 adult bees were collected from the brood nest area in August pre-treatment and in late January to determine dispersal phase mite density using the alcohol wash method^44^. Because samples used to determine mite density required a comparatively large number of bees, and bee colony size is important for winter survival^8^, those samples were restricted to pre-treatment in August and post winter at the end of the experiment. Just prior to placing hives in cold storage in August, and again in October, slick paperboard coated with petroleum jelly and covered with mesh were placed onto the hive floor to monitor Varroa mite fall within the hive^44^. The paperboards were removed after 3 days and the number of mites counted on each board.

### Gene expression analysis

For each colony and time point, pooled samples of 50 bees were homogenized in 8mL of ice cold phosphate buffered saline for three 12 s cycles at a speed of 4.5 m/s with a 15 s dwell between each cycle in an Omni Universal Bead Mill. RNA was extracted from 50uL of homogenate using a Maxwell RSC SimplyRNA Tissue Kit (Promega, USA) following the manufacturer’s protocol. cDNA synthesis was carried out using 1 µg of RNA and QuantiTect Reverse Transcription Kits (Qiagen, USA) according to the manufacturer’s protocols. Quantitative PCR (qPCR) was performed using a 1:10 dilution of cDNA to quantify transcript levels of *vitellogenin* (Vg), DWV-A and DWV-B. All reactions were carried out as follows: initial denaturation at 95 °C for 5 min; 40 cycles with denaturation at 95 °C for 10 s; an annealing temperature specific to each target (Vg = 58 °C, DWV-A = 60 °C and DWV-B = 62 °C) and extension temperature at 60 °C for 30 s. The reactions were carried out using Luna^®^ Universal qPCR Master Mix (New England Biolabs, USA) in triplicate on a CFX96 Real-Time PCR Detection System (Bio-Rad, USA). Relative expression levels were calculated based on standardized Ct values (ΔCt) using the geometric mean of honeybee *β-actin* and RP49 for normalization.

### Data analysis

Hive weight data were expressed as daily hive weight change from midnight to midnight. Temperature and CO_2_ concentration data were transformed into the daily average and the within-day detrended data, which was calculated as the difference between the 24 h running average and the raw data. Sine curves were fit to 3-day subsamples of detrended data using the cosinor() package in R^45^ to obtain amplitudes of in-hive temperature and CO_2_ data as a measure of parameter variability^35^. The average daily values and the amplitudes of each 3-day subsample for both temperature and CO_2_ concentration were used as response variables.

All response variables were examined for normality using Proc Univariate^46^, log transformed to improve normality if warranted, and then subjected to either: (1) a repeated-measures mixed-model MANOVA with treatment group (honey bee stock), day (or sampling occasion), year and all two-way interactions as fixed effects, a residual random effect and an autoregressive covariance structure ar(1) using Proc Glimmix; or (2) a non-parametric Wilcoxon test with a Dwass, Steel, Critchlow-Fligner (DSCF) multiple comparison analysis using Proc Npar1way^46^. Pre-treatment values for all response variables analyzed using parametric statistics were used as covariates to control for pre-existing conditions. The level of significance was set to α < 0.05. Pearson correlation coefficients between mite load and DWV levels were calculated. Changes in hive weight of 2 kg or more within a day (indicating high likelihood of error or a weather event), and days when colonies were fed, were removed.

## Supplementary Information

Below is the link to the electronic supplementary material.

Supplementary Material 1

## Data Availability

The data generated in this study, including continuous data on hive weight, temperature and CO2, hive evaluation data, and gene expression data, can be found at: Meikle, W., Ricigliano, V.A. (2025) Data from: Honey bee genetic resistance outperforms a cold-storage induced halt in brood production to control mites and viruses. Ag Data Commons. https://doi.org/10.15482/USDA.ADC/29998288.
