# Supplementary material for: Honey bee genetic resistance outperforms a cold-storage induced halt in brood production to control mites and viruses
Source: Sci Rep. 2026 Mar 21;16:11782. doi: 10.1038/s41598-026-44701-3 (PMC13066380; doi:10.1038/s41598-026-44701-3)
Supplement: Supplementary file 1 — Supplementary Material 1 [file 41598_2026_44701_MOESM1_ESM.pdf]

Honey bee genetic resistance outperforms a cold-storage induced halt in brood production to control mites and viruses

William G. Meikle[1]*, Milagra Weiss[1], Daniela Adjaye[2] and Vincent A. Ricigliano[3]

[1] Carl Hayden Bee Research Center, USDA-ARS, Tucson, AZ 85719, USA.
[2] Honey Bee Breeding, Genetics, and Physiology Research, USDA-ARS, Baton Rouge, LA 70820, USA.
[3] Invasive Species and Pollinator Health Research Unit, USDA-ARS, Davis, CA, USA 95616

*email: william.meikle@usda.gov

Supplementary Information

Table S1. Summary information for discrete data across bee stocks within treatment group: Adult bees, brood, relative expression of DWV-A, DWV-B and vitellogenin, and Varroa load per 100 bees and Varroa mite fall - averages and standard errors.

		CSU			Out		
**Parameter**	**Sample**	**Ital**	**Pol**	**Rus**	**Ital**	**Pol**	**Rus**
Adult wt	Pre-treat	2.75±0.31	2.57±0.22	2.60±0.19	2.83±0.27	2.61±0.25	2.48±0.21
kg	Post treat	2.17±0.20	1.83±0.18	1.83±0.10	2.30±0.33	2.19±0.27	2.34±0.26
	Pre winter	2.87±0.33	2.48±0.19	2.37±0.15	2.62±0.37	2.70±0.19	2.62±0.21
	Post winter	1.48±0.22	1.59±0.15	1.55±0.08	1.09±0.30	1.85±0.19	1.82±0.17
Brood area	Pre-treat	2039±111	1620±153	1488±110	2146±209	1373±100	1367±130
sq. cm	Post treat	79±31	39±29	5±4	2164±261	1784±267	1780±292
	Pre winter	1775±370	1618±201	1338±157	1387±270	1486±173	1215±210
	Post winter	289±100	208±82	213±57	377±117	193±42	142±75
DWV_A	Pre treat	3.7±2.4	4.2±1.4	12.8±3.9	0.9±0.4	2.7±1.8	0.4±0.3
rel. expr.	Post treat	5.0±2.6	0.1±0.1	0.9±0.9	145.5±105.1	0.5±0.5	3.3±3.3
	Post winter	7.8±6.1	0.0±0.0	10.5±10.5	2.7±1.1	0.0±0.0	0.0±0.0
DWV_B	Pre treat	19.0±8.7	37.7±21.1	35.8±8.8	55.6±35.0	32.6±21.4	1.8±1.1
rel. expr.	Post treat	24.1±11.5	4.3±3.8	14.5±9.5	823.0±429.7	18.2±13.5	1.7±1.7
	Post winter	31.4±25.4	0.5±0.5	20.6±17.1	53.5±28.6	2.2±1.6	0.0±0.0
Vg	Pre treat	0.3±0.0	0.4±0.1	0.2±0.0	0.2±0.0	0.4±0.1	0.3±0.0
rel. expr.	Post treat	0.7±0.2	0.8±0.1	0.7±0.1	0.4±0.1	0.6±0.1	0.8±0.1
	Post winter	0.2±0.1	0.3±0.1	0.2±0.0	0.3±0.1	0.2±0.0	0.2±0.1
Varroa	Pre treat	1.16±0.68	1.01±0.71	1.34±0.79	0.42±0.22	1.18±0.62	0.26±0.18
mite load	Post winter	3.05±0.84	1.05±0.34	1.30±0.52	4.18±1.20	1.36±0.49	0.87±0.34
Varroa	Pre treat	13.0±4.4	35.6±27.4	12.5±5.9	16.3±6.6	19.0±11.8	12.6±3.7
mite fall	Post treat	4.1±1.3	1.2±0.5	1.6±0.9	14.6±5.3	2.1±1.2	1.7±0.7

Table S2. Results of repeated measures MANOVA for log adult mass. “Num” is numerator; “Den” is denominator; “DF” is degrees of freedom; “Pre-treatment avg.” is average hive weight prior to cold storage as a covariate.

Effect	Num DF	Den DF	F Value	Pr > F
Treatment	1	47.37	1.38	0.2462
Queen	2	47.33	0.80	0.4557
Year	1	47.39	0.01	0.9258
Time	2	104.8	98.69	<0.0001
Treat * Queen	2	47.42	2.05	0.1400
Treat * Year	1	47.33	0.63	0.4331
Queen * Year	2	47.39	1.08	0.3492
Pre-treatment avg.	1	46.89	20.69	<0.0001

Table S3. Wilocoxon analysis of brood surface area. Shown are Dwass, Steel, Critchlow-Fligner (DSCF) multiple comparison analyses. “Ital” means Italian, “Pol” means Pol-line, and “Rus” means Russian; “P” indicates probability of the statistic.

	CSU vs out			Ital vs Pol		Ital vs. Rus		Pol vs. Rus	
Sample	Chi-sq.	P	Within treat	Z	P	Z	P	Z	P
Pre	0.645	0.422		**3.616**	**0.001**	**4.011**	**<0.001**	0.394	0.918
1	**38.23**	**<0.001**	CSU	1.139	0.490	2.000	0.112	0.710	0.758
			out	0.653	0.791	0.815	0.693	-0.132	0.990
2	0.698	0.403	CSU	0.267	0.962	1.155	0.480	1.285	0.404
			out	-0.163	0.985	0.490	0.876	0.927	0.623
3	0.018	0.893	CSU	0.455	0.892	0.816	0.693	0.312	0.948
			out	1.021	0.564	1.554	0.266	1.283	0.405
4	0.072	0.789	CSU	-0.178	0.983	1.732	0.193	1.225	0.439
			out	-2.075	0.095	-1.810	0.166	-0.044	0.999

Table S4. Wilcoxon analysis of relative expression of DWV-A and DWV-B virus; shown are main effects comparisons of bee stock. Shown are Dwass, Steel, Critchlow-Fligner (DSCF) multiple comparison analyses. “Ital” means Italian; “Pol” means Pol-line; and “Rus” means Russian; “P” indicates probability of the statistic.

	**Ital vs Pol**		**Ital vs. Rus**		**Pol vs. Rus**	
**Sample**	**Z**	**P**	**Z**	**P**	**Z**	**P**
DWV-A Pre-treat	0.746	0.736	0.288	0.955	-0.912	0.632
DWV-A Post-treat	**2.112**	**0.039**	**2.531**	**0.031**	1.353	0.366
DWV-A Post-winter	**2.442**	**0.039**	**2.629**	**0.023**	0.587	0.827
DWV-B Pre-treat	0.593	0.824	0.564	0.836	-0.168	0.935
DWV-B Post-treat	**2.442**	**0.039**	**2.689**	**0.020**	0.264	0.962
DWV-B Post-winter	**3.235**	**0.004**	**3.026**	**0.007**	0.552	0.845

Table S5. Wilcoxon analysis of relative expression of DWV-A and DWV-B virus; shown are main effects comparisons of cold storage treatment and year of study. Shown are Dwass, Steel, Critchlow-Fligner (DSCF) multiple comparison analyses. “CSU” means cold storage unit treatment; “out” means no cold storage treatment; “P” indicates probability of the statistic.

	**CSU vs out**		**2023 vs 2024**	
**Sample**	**Chi-sq.**	**P**	**Z**	**P**
DWV-A Pre-treat	1.030	0.310	**3.526**	**0.001**
DWV-A Post-treat	0.046	0.831	-1.930	0.059
DWV-A Post-winter	0.142	0.706	2.010	0.050
DWV-B Pre-treat	2.201	0.138	**2.639**	**0.011**
DWV-B Post-treat	0.304	0.581	1.3385	0.186
DWV-B Post-winter	0.753	0.385	1.1512	0.255

Table S6. Wilcoxon analysis of relative expression of DWV-A and DWV-B virus; shown are nested effects of bee stock within treatment group. Shown are Dwass, Steel, Critchlow-Fligner (DSCF) multiple comparison analyses. "CSU" means cold storage unit treatment; "out" means no cold storage treatment; "Ital" means Italian; "Pol" means Pol-line; and "Rus" means Russian; "P" indicates probability of the statistic.

Factor	Sample	Within treat	Ital vs Pol		Ital vs. Rus		Pol vs. Rus	
			Z	P	Z	P	Z	P
DWV-A	Pre	CSU	1.330	0.857	0.775	0.997	-1.061	0.730
		out	0.529	0.379	-0.076	0.719	-0.756	0.538
	1	CSU	1.955	0.722	2.123	0.540	1.347	0.949
		out	0.770	0.124	1.059	0.085	0.309	0.369
	2	CSU	1.104	0.055	2.163	0.289	1.634	0.573
		out	2.309	0.512	1.505	0.078	-1.006	0.232
DWV-B	Pre	CSU	0.680	0.775	-0.151	0.988	-1.210	0.448
		out	0.798	0.704	1.901	0.138	0.817	0.693
	1	CSU	1.422	0.330	0.962	0.601	-0.327	0.943
		out	1.715	0.200	**2.776**	**0.015**	1.280	0.406
	2	CSU	**2.666**	**0.021**	1.155	0.480	-1.470	0.306
		out	2.252	0.063	**2.958**	**0.009**	1.810	0.166

Table S7. Results of repeated measures MANOVA for relative expression of *vitellogenin* (Vg), transformed as the arcsine of the square root. “Num” is numerator; “Den” is denominator; “DF” is degrees of freedom; “Pre-treatment avg.” is *vitellogenin* prior to cold storage as a covariate.

**Effect**	**Num DF**	**Den DF**	**F Value**	**Pr > F**
Treatment	1	40.4	2.48	0.1228
**Queen**	**2**	**39.45**	**7.08**	**0.0024**
**Year**	**1**	**39.3**	**27.07**	**<0.0001**
Time	1	50.78	67.88	<0.0001
Treat * Queen	2	39.74	1.57	0.2215
Treat * Year	1	40.46	0.13	0.7233
Queen * Year	2	40.32	0.38	0.6875
Pre-treatment avg.	1	42.99	0.60	0.4444

Table S8. Wilcoxon analysis of the results of alcohol wash to determine Varroa mite load; including main effects comparisons of cold storage treatment and bee stock, and effects of bee stock nested within treatment. Shown are Dwass, Steel, Critchlow-Fligner (DSCF) multiple comparison analyses. “CSU” means cold storage unit treatment; “out” means no cold storage treatment; “Ital” means Italian; “Pol” means Pol-line; and “Rus” means Russian; “P” indicates probability of the statistic.
nonparametric analysis; “Difference” means the difference between the pre-treatment and post winter samples

	**CSU vs out**			**Ital vs Pol**		**Ital vs Rus**		**Pol vs. Rus**	
**Sample**	**Chi-sq.**	**P**	**Within treat**	**Z**	**P**	**Z**	**P**	**Z**	**P**
Pre-treat	0.102	0.749		0.129	0.991	0.624	0.807	0.357	0.932
Post winter	0.003	0.958		**2.953**	**0.009**	**3.160**	**0.005**	0.524	0.860
Post winter			CSU	2.139	0.082	1.833	0.159	0.083	0.996
Post winter			out	2.077	0.095	**2.345**	**0.050**	0.716	0.754
Difference	0.348	0.556		**2.967**	**0.009**	**3.039**	**0.007**	-0.092	0.995
Difference			CSU	1.603	0.245	1.829	0.160	0.247	0.967
Difference			out	**2.430**	**0.040**	**2.430**	**0.040**	-0.444	0.897

Table S9. Wilcoxon analysis of the results of mite fall to estimate relative Varroa population size; including main effects comparisons of cold storage treatment and bee stock, and effects of bee stock nested within treatment. Shown are Dwass, Steel, Critchlow-Fligner (DSCF) multiple comparison analyses. "CSU" means cold storage unit treatment; "out" means no cold storage treatment; "Ital" means Italian; "Pol" means Pol-line; and "Rus" means Russian; "P" indicates probability of the statistic. nonparametric analysis

	**CSU vs out**			**Ital vs Pol**		**Ital vs Rus**		**Pol vs. Rus**	
**Sample**	**Chi-sq.**	**P**	**Within treat**	**Z**	**P**	**Z**	**P**	**Z**	**P**
Pre-treat	0.033	0.857		0.334	0.940	0.335	0.940	-0.015	1.000
Post treat	0.085	0.770		2.154	0.079	2.207	0.070	0.196	0.979
Post treat			CSU	2.153	0.080	2.302	0.056	1.136	0.492
Post treat			out	1.231	0.435	0.914	0.631	-0.589	0.826
Difference	0.951	0.329		1.277	0.408	1.535	0.275	0.248	0.967
Difference			CSU	0.044	0.999	-0.178	0.983	-0.340	0.938
Difference			out	1.757	0.184	2.041	0.103	0.751	0.733

Table S10. Results of repeated measures MANOVA for daily hive weight change (see text for details). “Num”=numerator; “Den”=denominator; “DF”=degrees of freedom; “Pre-treatment avg.” is hive weight prior to cold storage as a covariate.

**Effect**	**Num DF**	**Den DF**	**F Value**	**Pr>F**
Treatment	1	1363	1.44	0.2305
**Queen**	**2**	**1363**	**9.72**	**<0.0001**
**Year**	**1**	**1378**	**6.31**	**0.0121**
Time	163	8314	18.99	<0.0001
Treat * Queen	2	1363	0.63	0.5308
Treat * Year	1	1363	0.74	0.3885
Queen * Year	2	1363	2.05	0.1297
Pre-treatment avg.	1	1363	0.75	0.3858

Table S11. Results of repeated measures MANOVA for log average daily hive temperature and daily hive temperature amplitudes (see text for details). "Num" is numerator; "Den" is denominator; "DF" is degrees of freedom; "Pre-treatment avg." and "Pre-treatment ampl." are hive temperature average or amplitude, respectively, prior to cold storage as a covariate.

Response variable	Effect	Num DF	Den DF	F Value	Pr>F
Log average daily temperature	**Treatment**	**1**	**148.7**	**5.09**	**0.0255**
	Queen	2	148.7	2.22	0.1127
	Year	1	148.9	0.88	0.3484
	Time	164	8723	22.51	<0.0001
	**Treat * Queen**	**2**	**148.7**	**3.65**	**0.0285**
	Treat * Year	1	148.8	0.54	0.4638
	Queen * Year	2	148.8	2.50	0.0852
	Pre-treatment avg.	1	148.8	2.50	0.0852
Log daily temperature amplitude	**Treatment**	**1**	**99.55**	**4.66**	**0.0333**
	Queen	2	99.53	0.13	0.8793
	**Year**	**1**	**99.59**	**4.62**	**0.0340**
	Time	53	2821	16.94	<0.0001
	Treat * Queen	2	99.54	1.73	0.1834
	Treat * Year	1	99.53	0.81	0.3692
	Queen * Year	2	99.56	2.77	0.0673
	Pre-treatment ampl.	1	99.3	2.73	0.1019

Table S12. Results of repeated measures MANOVA for log average daily hive CO_2 concentration and daily hive CO_2 concentration amplitudes (see text for details). "Num"=numerator; "Den"=denominator; "DF"=degrees of freedom; "Pre-treatment avg." and "Pre-treatment ampl." are hive CO_2 concentration average or amplitude, respectively, prior to cold storage as a covariate.

Response variable	Effect	Num DF	Den DF	F Value	Pr>F
Log average daily CO_2 concentration	Treatment	1	276.3	0.08	0.7809
	Queen	2	276.2	2.65	0.0722
	**Year**	**1**	**276.6**	**36.33**	**<0.0001**
	Time	163	8559	28.92	<0.0001
	**Treat * Queen**	**2**	**276.2**	**3.25**	**0.0401**
	Treat * Year	1	276.3	1.37	0.2420
	**Queen * Year**	**2**	**276.3**	**11.66**	**<0.0001**
	Pre-treatment	1	275.9	5.50	0.0198
Log daily CO_2 amplitude	Treatment	1	216.8	1.37	0.2438
	Queen	2	216.8	2.80	0.0628
	**Year**	**1**	**217**	**20.32**	**<0.0001**
	Time	54	2827	12.05	<0.0001
	**Treat * Queen**	**2**	**216.8**	**3.32**	**0.0379**
	Treat * Year	1	216.8	0.22	0.6362
	**Queen * Year**	**2**	**216.9**	**3.20**	**0.0428**
	Pre-treatment	1	216.4	0.66	0.4172
